# Income-Related Inequality in Traffic Accident Health Outcomes (Injury, Disability and Mortality): Evidence from the Nationwide Survey in Iran

**Published:** 2020-04

**Authors:** Payam ROSHANFEKR, Mohammad-Reza KHODAIE-ARDAKANI, Homeira SAJJADI, Hossein MALEK AFZALI ARDAKANI

**Affiliations:** 1. Social Determinants of Health Research Center, University of Social Welfare and Rehabilitation Sciences, Tehran, Iran; 2. Department of Epidemiology and Biostatistics, School of Public Health, Tehran University of Medical Sciences, Tehran, Iran

**Keywords:** Traffic accidents, Crush injury, Socioeconomic factor, Inequality, Income

## Abstract

**Background:**

Despite many efforts, Iran continues to have a high rate of traffic accidents and poor health outcomes. This study aimed to measure income-related inequality for traffic accident health outcomes in Iran, a country with one of the highest rates of traffic accidents and related health problems.

**Methods:**

The source of data was a national representative survey named the Iranian Multiple Indicator Demographic and Health Survey (IrMIDHS, 2010). Monthly household income is obtained through self-report in different quarters. Disparity rate ratio (DRR), slop index of inequality (SII) and the population attributable risk percentage measure (PAR%) were calculated. The concentration index (CI) of RTIs was used as our measure of socioeconomic inequality and decomposed into its determining factors.

**Results:**

Using the DRR index, in the lowest income group, the risk of death from an accident was 2.3 times, greater and the risk of accidental disability was 11.7 times greater than for the third income quartet. The slope index also shows that the rate of road traffic deaths, disability and injury per 100,000 individuals decreased by 28, 82, and 392 moving from lower to higher incomes. This decrease in injury was about 581 for motorcyclists. CI was −0.04078643 (SE=.01424828, *P-*value 0.004). Male sex (68.9%), 15–29 yr old age (9.4%), employed activity status (20.8%) has a positive contribution in the RTIs concentration index.

**Conclusion:**

In addition to intervention related to the road safety and vehicles and reducing human errors, prevention of the road traffic ill health outcomes requires attention to reduction of inequality in society.

## Introduction

Health outcomes from traffic accidents are a major issue affecting global health (1), especially in developing countries, which are rapidly becoming motorized (2). About 90% of road traffic deaths occur in low- and middle-income countries, although these countries have only 54% of the total number of vehicles globally (3). Injuries and accidents are a major cause of mortality and disability and a major contributor to the health gap of society (4). The death rate from accidents in low-income and middle-income countries is 3.4-fold higher than in high-income countries (5). In the context of the association of income inequality with health outcomes, evidence exists, especially in developed countries (6, 7), however, research on the relationship between income inequality and these outcomes in other countries is limited (8).

Although much is spent on public health, it is not responsive to public health needs. The removal of inequality and prevention of injury would save more than half a million lives globally and reduce costs or make budgeting more effective (9).

The prevalence of adverse road traffic health outcomes is higher among poor people (10, 11) and they experience more exposure to risk factors for various reasons, including living in high-risk environments and conditions(12). Moreover, in this group, risky behaviors including traffic-related risky behaviors are more common(13). Among the factors that increase the risk of disability or death in low-income groups and residents of poor areas (where these people live), their transfer from the scene to health centers can be mentioned (14).

Iran is a developing country that has advanced various plans and measures in the last decade regarding road safety (15). Yet Iran continues to have a high rate of traffic accidents and poor health outcomes (16–18). The cost of road traffic accidents is about 2.19% the gross domestic product of the country (19). Researchers have demonstrated a relationship between income gap and traffic mortality risk in metropolitan areas of Iran (10), but population-based research at the country scale has not been addressed.

The purpose of this study was to investigate the inequalities associated with the income gap on the health outcomes of traffic accidents in Iran based on data from a national population survey. These findings can help health and social welfare policy-makers obtain more support to reduce inequality in order to improve the health and quality of life of the citizens.

## Methods

The data was derived from the national cross-sectional survey Iran Multidimensional Demographic and Health Survey (IrMIDHS, 2010). The study was designed to obtain a widespread and diverse image of societal health and assess the socio-demographic characteristics of individuals to determine the effect of social factors on health and health inequity (20). Sampling was done using a multi-stage stratified cluster sampling method in 2187 urban clusters and 909 rural clusters in 31 provinces of the country. The data from resident households (Iranian or immigrant) was collected through face-to-face interviews by filling out questionnaires about households (107 questions), children under the age of 5 (88 questions) and women (145 questions). The questionnaire was based on similar global and national studies and was validated by an expert panel. The response rate was 95%, with data from 111415 live individuals and 430 deaths in the study year.

The data was collected on 1354 traffic injuries, 43 deaths and 34 cases of disability within one year. To assess the socio-economic status, a question on monthly household income divided the response into income groups of less than 2.5 million, 2.5 to 5 million, 5 to 10 million, 10 to 20 million and more than 20 million Iranian rials (IRR). In the analysis, the two highest income groups were combined and analyzed as a quartet. The response rate was 91.03%.

The inequality indices of the measurement of effect, disparity rate ratio (DRR) and rate difference index (RDI) were used to calculate the measure of association. Population attributed risk (PAR) was used as a measure of potential impact and the slope index of inequality(SII) as a measure of the regression of socioeconomic status rankings with health outcomes (21, 22).

The concentration index was used as the measure of socioeconomic inequality in road traffic injuries. It can be computed as twice the (weighted) covariance of the health variable and a person’s relative rank in terms of economic status, divided by the variable mean according to Equation [5] (23, 24).

[1] $$C=\frac{2}{\mu }COVw\left(yi,Ri\right)$$

The value of the concentration index can vary between-1 and +1. Its negative values imply that a variable is concentrated among disadvantaged people while the opposite is true for its positive values. When there is no inequality, the concentration index will be zero (25). Null hypotheses are the indices are equal to zero (no inequality) The method proposed by Wagstaff et al. (26, 27) showed in formula [6] and its a modification for binary outcomes (26, 28) was used to decompose socioeconomic inequality in RTIs into its determinants. A decomposition analysis allows one to estimate how determinants proportionally contribute to inequality (e.g. the gap between poor and rich) in a health variable.

[2] $$CI=\sum_{k}^{n}\left(\frac{\beta \text{k}\bar{\text{X}}\text{k}}{\mu }\right)\text{CK}+\frac{\text{GC}ɛ}{\text{μ}}=\text{C}\hat{\text{y}}+\frac{\text{GC}ɛ}{\text{μ}}$$

All explanatory variables (Determinants) were included as categorical—dummy—variables.

Extracted data were analyzed using SPSS -PC Ver. 18.0 (Chicago, IL, USA) and released version of stata.12 (1985-2011 LP STATA Corp, Texas USA) and the Distributive Analysis STATA Package (DASP) software.

The ethical consideration in study proposal and procedures were reviewed and approved by the Ethics Committee and the Research Review Board at University of social welfare and rehabilitation Sciences (USWRS).(IR.USWR.REC.1394.189). More data about sampling data gathering and ethical issues of IrMIDHS was discussed comprehensively in the study protocol article (20).

## Results

Data from 111782 individuals in the country sample showed that 43 deaths, 34 cases of disability and 1538 injuries related to road traffics accidents occurred in the year prior to the study. Per 100000 individuals, for 10 deaths, 7.9 disabilities and 357.7 injuries, this computes to an annual prevalence of 38.5, 30.4 and 1375.9, respectively. Overall, 91.07% of respondents reported their monthly household income and 8.93% failed to report it (the option “I do not know” in the questionnaire). Of these, 31.28% of respondents reported an income of less than 2.5 million (59.37 USD); 47.29% of 2.5 to 5 million (59.37 to 118.75 USD); 18.25% of 5 to 10 million(118.75 to 237.5 USD) and 3.18% of over 10 million IRR (1 million IRR=23.75 USD)

The distribution of the quartets from the lowest to the highest income group is Q1=31.3%; Q2=47.3%; Q3=18.2%; Q4=3.2%. Table 1 shows the income-related inequality indicators for different traffic accident outcomes.

**Table 1:** Income-related inequality in traffic accident health outcomes (injury, disability and mortality) in Iran

***Income quartile***	***population***	***RTM***	***RTD***	***RTI***	***PRTIs***	***VRTIs***	***MRTIs***	***RTM per100000***	***RTD per100000***	***RTI per100000***	***Pedestrian per 100000***	***vehicle per 100000***	***motorcycle per 100000***
Q1	31,839	12	20	496	76	144	256	37.69	62.82	1557.84	238.70	452.28	804.05
Q2	48,125	19	10	650	80	271	270	39.48	20.78	1350.65	166.23	563.12	561.04
Q3	18,571	3	1	249	47	113	82	16.15	5.38	1340.80	253.08	608.48	441.55
Q4	3,240	0	0	33	4	25	3	0	0.00	1018.52	123.46	771.60	92.59
	101,775	34	31	1,428	207	553	611	33.41	30.46	1403.10	203.39	543.36	600.34
Response Rate	91.3%					DRR		2.33 ^*^	11.67 ^*^	1.53	1.93	0.59	8.68
						RDI		37.7	62.8	539.3	115.2	−319.3	711.5
						PAR%		100 [51.6 ^†^ ]	100 [82.3 ^†^ ]	27.4	39.3	−42.0	84.6
						SII		−28.27	−82.52	−392.74	−34.65	255.33	−581.54

RTM: Road Traffic Mortality; RTD: Road Traffic Disability; RTI: Road Traffic Injury; PRTI: Pedestrian’s Road Traffic Injury; VRTI: Vehicle’s Road Traffic Injury; MRTI: Motorcycle’s Road Traffic Injury; DRR: Disparity Rate Ratio; RDI: Rate Difference Index; PAR: Population Attributed Risk; SII: Slop Index of Inequality

* Outcome ratio in Q1 and Q3;

† difference between outcome in Q1 and Q3 compared source: from the Multiple Indicator Demographic and Health Survey (IrMIDHS, 2010)

### DRR index

The households in the lowest income quartet (Q1) had a 2.33 times greater risk of death and an 11.67-fold greater risk of disability than those in the higher income quartet (Q3). The risk of injury among the households of the lowest income quartet was 1.53 higher than that of households with the highest income quartet. The risk of injury to pedestrians in the first quarter was 1.93 times higher than for the fourth quartet. For disability, this risk was 8.68 times higher. The risk of injury in the car accident increased in the higher income groups.

### RDI index

The difference in the risk of disability and injury in the upper and lower quartets was 62.8 and 539.3 per 100000 individuals, respectively. The difference for injury was 115.2 for pedestrians and 711.5 for motorcyclists per 100000 individuals.

### PAR index

If all the households in the country were to have the same rate of road traffic incidents (RTIs) as those in the best socioeconomic status (high-income quartet), the road traffic injuries would be reduced by 27.4% in the general population, 39.3% for pedestrians and 84.6% for motorcyclists.

### Slope index

The number of road traffic deaths per 100,000 individuals decreased by 28 cases, disability by 82 cases and injury by 392 cases moving from the lowest to the highest income groups. This decrease is about 581 for motorcyclists.

### Concentration Index (CI)

**Fig. 1:**
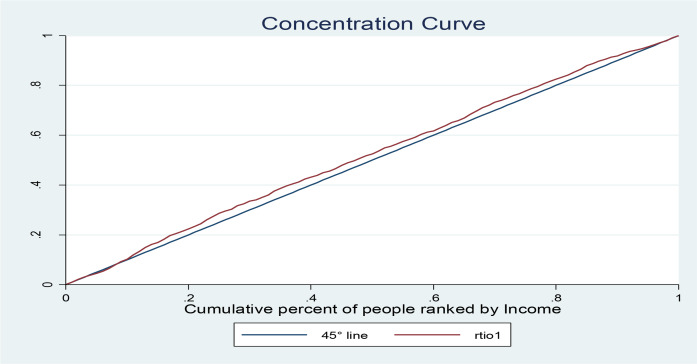
The concentration curve of RTIs in Iran (IrMIDHS,2010)

In order to quantify the extent of this inequality in RTIs in Iran, overall concentration indices were −0.04078643 (SE=.01424828, *P*-value 0.004) the concentration curve are presented in Fig. 1. The results show that significant inequalities are observed.

### Decomposition of CI in RTI

**Fig. 2:**
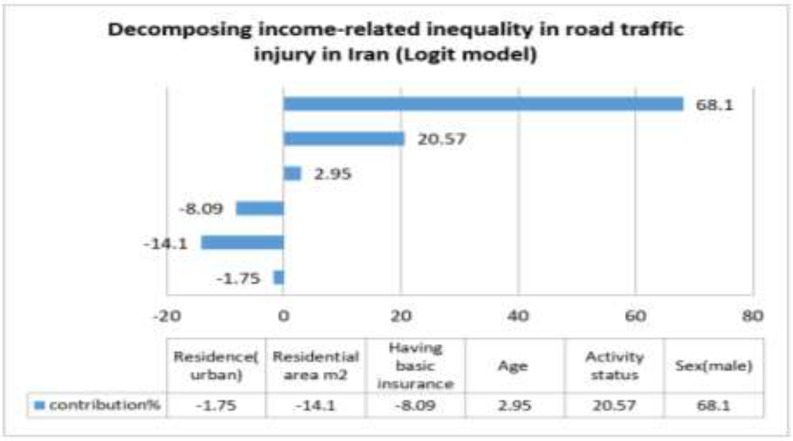
Graphical Representations of CI and Decomposition Analysis of RTIs into its determinants (IrMIDHS, 2010)

Socio-demographic characteristics and reported RTIs among different subgroups are shown in Table 2. The results show that in the year leading to study 79.2% of the people injured from road traffic accidents are male, 67.9% live in rural areas, 43.9% are 15–29 yr old, 76.1% are employed or looking for a job, 77.7% have basic insurance and 45.5% are in second income quantile (Q1 is poorer). The adjusted associations between RTIs and its determinants in Iran and decomposition components are shown in Table 3. We can see that male sex (68.9%), 15–29 yr old age (9.4%), employed activity status (20.8%) have a positive contribution to RTIs concentration index. Graphical Representations of CI and Decomposition Analysis are shown in Fig. 2.

**Table 2:** Values of Inequality in Road traffic injury Based on household income per month

***Variable***	***Socio demographic Characteristic N (%)***	***Injured by road traffic accident N (%)***	***Not involved in road traffic accident N (%)***
Place of residence			
Urban	76155 (68.10)	1044 (67.88)	74837 (68.11)
rural	35670 (31.90)	494 (32.12)	35040 (31.89)
Sex			
Male	56849 (51.02)	1218 (79.19)	56631 (50.63)
Female	54566 (48.99)	320 (20.81)	54246 (49.37)
Age			
<15	26599 (24.28)	183 (11.9)	26416 (24.45)
15–29	33907 (30.95)	676 (43.95)	33231 (30.76)
30–45	26060 (23.78)	411 (26.72)	25649 (23.74)
46–59	14644 (13.36)	189 (12.29)	14455 (13.38)
> 60	8360 (7.63)	79 (5.14)	8281 (7.67)
Activity status			
Employed (looking for)	46661 (55.53)	1035 (76.1)	45626 (55.19)
Housekeeper	27074 (32.22)	156 (11.47)	26918 (32.56)
Student(school or college)	10291 (12.25)	169 (12.43)	10122 (12.24)
Residential area			
< 65	24464 (24.22)	370 (27.05)	24094 (24.18)
65–95	26255 (25.99)	335 (24.49)	25920 (26.01)
96–125	25170 (24.92)	336 (24.56)	24834 (24.92)
> 125	25116 (24.87)	327 (23.9)	24789 (24.88)
Having Basic insurance			
No	20394 (18.32)	343 (22.33)	20051 (18.26)
Yes	90923 (81.68)	1193 (77.67)	89730 (81.74)
Income per month			
(<59.37USD)Q1	31718 (31.26)	496 (34.73)	31222 (31.22)
(59.67–118.75 USD)Q2	47986 (47.3)	650 (45.52)	47336 (47.33)
(118.75–237.5USD)Q3	18510 (18.25)	249 (17.44)	18261 (18.26)
(>237.5USD)Q4	3236 (3.19)	33 (2.31)	3203 (3.20)

**Table 3:** Decomposition analysis of concentration index of Roar traffic injuries ranked by income groups (IrMDHS,2010)

***Determinants***	***Coefficient***	***Mean***	***Elasticity***	***Concentration index(C)***	***Contribution to C***	***Contribution to C (%)***	
Residence(urban)	−0.071	0.305	−1.34844	−0.05281	0.071205	−1.74566	−1.75
Sex(male)	1.275	0.492	38.94879	−0.07132	−2.7778	68.09995	68.1
Age (yr)							
15–29	0.249	0.397	6.128831	−0.06268	−0.38416	9.417878	
30–45	−0.005	0.313	−0.10638	−0.04113	0.004376	−0.10727	
46–59	−0.148	0.173	−1.58937	−0.04602	0.073143	−1.79317	
> 60	−0.506	0.094	−2.95256	−0.06315	0.18646	−4.57122	2.95
Activity status							
Employed	0.273	0.557	9.433862	−0.08992	−0.84827	20.79603	
student	0.048	0.123	0.370472	0.024702	0.009152	−0.22436	20.57
Residential area							
65–95	−0.259	0.259	−4.17532	−0.07031	0.293551	−7.19664	
96–125	−0.225	0.253	−3.5385	−0.01397	0.049444	−1.21215	
> 125	−0.207	0.259	−3.33498	−0.06962	0.23219	−5.69232	−14.10
Having basic insurance	−0.183	0.831	−9.43998	−0.03496	0.330007	−8.0904	−8.09
C	−0.04079						67.68
ɲ	0.016102						

## Discussion

The findings of this study showed inequality in the distribution of road ill-health outcomes in traffic accidents according to income in Iran. The data also indicated an increase in inequality in the more severe outcomes (death and disability) in comparison with injury. The distribution of these forms of inequality was not the same for all road user groups. The ratio of inequality for pedestrian victims between the highest and the lowest income quartets was less than 1.5 times. For motorcycle RTIs, it was up to 15 times higher. More information about the distribution of death and disability in subgroups of users could not be extracted from the IrMDHS data.

Other research has confirmed a relationship between income inequality and the road traffic health outcomes (11, 12, 29, 30) as well as different levels of risk in different road user groups (31). In Fars Province in Iran, the risk of morbidity and mortality of motorcyclists were about tenfold that of those who travel in vehicles (32). WHO classified motorcyclists as a high-risk group in this regard (3, 5). A review of relevant resources in Iran over the past two decades has shown that motorcyclists often have little education and rank in the lower socioeconomic groups (33). In Sweden, the difference in income inequality and traffic accident outcomes had been confirmed by various user groups (34). In the case of pedestrians, a study in the state of California showed that being from a low-income family (below 185% of the federal poverty level) was the strongest predictor of pedestrian injury and that a 1% increase in the low-income group will increase pedestrian accidents by 2.8% (7).

The prevalence of adverse traffic accident health outcomes is higher among low-income groups and that they experience greater exposure to risk factors because they tend to live in high-risk environments and conditions. Studies have shown that, in this group, risky behavior, including traffic-related risky behavior, is more common (13). In Iran, the prevalence of accidents and related mortality is much higher among low-income groups (10). A cohort study in Sweden showed that young motorcyclists in the lower-income strata are more at risk for moderate and severe traffic injuries (35). In Spain, people with lower socioeconomic status face increased stress in their everyday lives, which can negatively affect their focus on driving (36).

Among the factors that increase the risk of disability or death in low-income groups and residents of poor areas is their mode of transfer from the scene to a health center. In most low-income and middle-income countries (LMICs), traffic accident victims are transported to health centers by relatives, taxi drivers, truck drivers, police officers or other drivers who do not usually have necessary medical training and this can intensify their injuries (14). Low-income residents also usually have access problems (37) to track their traffic accident-related health problems either because they live in less prosperous areas and have no or little access to health care facilities (38, 39) or because they are not able to afford the cost of the most appropriate services. Inadequate public health infrastructure and poor access to health services are important reasons for high rate of RTIs injuries or their increased severity (40).

There is more evidence about treatment and benefitting from hospital services for communicable diseases (41) than for traffic accidents, but which indicates that low-income individuals are less likely to follow up their treatment (42) and are likely to postpone or cancel post-injury follow-up and care that may exacerbate of their ill-health (43). With regard to disability, global evidence suggests that low-income or jobless individuals or those with little education are at increased risk of disability (44, 45). In China, concentration index had shown that a negative value (−0.192) means that adults in a disadvantaged financial situation suffer more from disabilities after traffic accidents (22). Harmful outcomes related to income inequality should not be imposed on the poor and that all should pay the cost of reducing illness and crime in society, as they will all suffer from a decrease in the quality of civil society and social capital (46).

A limitation of this study relates to the nature of the indicators used to illustrate the health-related inequality discussed in various sources (21, 47). The method of income measurement in this study, the self-report method, based on income quartets, is not sensitive to some intra-group differences and may create problems such as low reporting and recall bias.

## Conclusion

In addition to intervention related to road safety and vehicles and reducing human errors, prevention of the road traffic ill-health outcomes requires attention to reduction of inequality in society.

## Ethical considerations

Ethical issues (Including plagiarism, informed consent, misconduct, data fabrication and/or falsification, double publication and/or submission, redundancy, etc.) have been completely observed by the authors.
